# Relationship between objective measures of physical activity and weather: a longitudinal study

**DOI:** 10.1186/1479-5868-3-21

**Published:** 2006-08-07

**Authors:** Catherine B Chan, Daniel AJ Ryan, Catrine Tudor-Locke

**Affiliations:** 1 Department of Biomedical Sciences, University of Prince Edward Island, Charlottetown, PE, Canada; 2 Department of Mathematics and Statistics, University of Prince Edward Island, Charlottetown, PE, Canada; 3 Walking Research Laboratory, Department of Exercise and Wellness, Arizona State University East, Mesa, AZ, USA

## Abstract

**Background:**

The weather may be a barrier to physical activity but objective assessment of this hypothesis is lacking. Therefore we evaluated the effect of temperature, rain or snow, and wind speed on the daily physical activity of adults.

**Methods:**

This report contains data from 25 males (BMI (mean ± SD): 28.7 ± 3.83 kg/m^2^) and 177 females (BMI: 29.2 ± 5.92 kg/m^2^) enrolled in an intervention to increase physical activity. Steps/day of the participants was measured by pedometer. Weather data were obtained from Environment Canada. A total of 8,125 observations were included in a mixed linear model analysis.

**Results:**

Significant weather related variables (at the 5% level) impacting steps/day included: seasonal effects related to the interaction between weekday and month; mean temperature, total rainfall, interactions between gender, BMI and total snow, interactions between maximum wind speed and BMI, and the amount of snow on the ground. The estimated magnitudes for the various effects were modest, ranging from ~1% to ~20%. Thus for an average individual taking ~10,000 steps/day, weather-dependent changes in physical activity could reach 2,000 steps/day.

**Conclusion:**

We conclude that weather had modest effects on physical activity of participants in an intervention to increase their activity. It should be stressed that these effects may be different for less or more motivated people. With this in mind, we suggest that the effect of weather on physical activity in the general population needs to be objectively assessed to better understand the barrier it poses, especially as it relates to outdoor recreation or work activities.

## Background

The weather has been suggested as an environmental factor affecting physical activity [1]. Using surveys, Humpel *et al*. [2] showed that weather was least inhibiting for individuals who walked specifically for exercise or who were "high neighborhood walkers". Neighborhood walkers were defined as individuals who walked for any reason in and around their neighborhood. Similarly, people who found exercise most enjoyable were least likely to cite the weather as a barrier [3]. Also using self-report, inclement weather was identified as a barrier to becoming physically active in analyses of women [4-7]. Weather or time of year apparently impacts all socioeconomic groups as a barrier to physical activity [8]. However, not all studies using self-report of physical activity found associations with weather [9,10]. Moreover, the magnitude of the impact of weather may not have been accurately assessed because neither physical activity nor the weather was measured objectively.

There have been only two reports of the effects of weather on physical activity where activity was measured objectively. In one case, steps/day measured by pedometer in 23 individuals over a one-year period were found to vary with the season (summer > winter) for individuals in South Carolina and Tennessee [11]. Another study of 41 elderly Japanese found an exponential decrease in physical activity with increasing precipitation (rain) while the activity increased as temperature became warmer, up to 17°C, then declined [12]. In neither case was snow a significant factor in the weather.

Although physical activity is inversely proportional to BMI [13,14] it is not known to what extent this relationship may be influenced by weather. To our knowledge, there are no reports of the effects of weather on physical activity when body mass index (BMI) is a considered variable. Previously, the relationship between weather as a barrier to physical activity was shown to be similar in men and women [2]. In the current study, we compared physical activity measured by pedometer with objective weather data in a longitudinal study design to determine if day-to-day variation in weather (considering also the season and day of week) in conjunction with BMI and gender had a significant impact on physical activity. The weather variables assessed included temperature, rain or snow, and maximum wind speed.

## Research methods and procedures

### Participants and data collection

Physical activity data (steps/day) were collected from a total of 203 adults (25 men and 178 women) enrolled in a facilitated intervention (the First Step Program (FSP)) that utilized pedometers to provide feedback and motivation to increase physical activity. The FSP, based on Social Cognitive Theory, is a facilitated behavioural intervention that utilizes goal-setting, self-monitoring and feedback using a pedometer [15]. Participants met in small groups with trained facilitators for 4 weeks, then continued goal-setting and self-monitoring independently for 8 weeks. Steps/day were recorded daily in a logbook. After 12 weeks, participants met with facilitators to complete measurements and hand in logbooks. The study was approved by the Research Ethics Board of the University of Prince Edward Island. An important facet of the intervention was that each participant was encouraged to set individualized goals for their daily physical activity; in general, the goals increased from week 1–4 and then levelled off [16]. In addition, we found that the time to plateau at a new activity level averaged ~4 weeks [17]. The intervention was delivered to the participants either in workplace (n = 156, BMI = 29.3 ± 6.0 kg/m^2^) or community settings (n = 47, BMI = 28.6 ± 4.3 kg/m^2^; differences in BMI not significantly different between workplace and community groups). Peer facilitators in both contexts received similar training from program directors and followed a specified curriculum at each meeting. Details of the participants and the intervention in the workplace setting have been published elsewhere [13,17]; in that study, the workplaces were selected because of largely sedentary job descriptions (eg., clerical, administrative). Participants were recruited by methods acceptable to each workplace (eg., email or paper bulletins). The majority of the participants were relatively inactive, with a baseline steps/day of 7,469 ± 3,460 for females and 8,708 ± 2,684 for males (overall, 7,627 ± 3,390 steps/day). Participants in the community programs were recruited by radio and newspaper advertisements but were, on average, not more active (8,089 ± 3,306 steps/day, p = 0.291). At baseline, the participants provided basic demographic (gender, age, education, employment) and health information (history of smoking, type 2 diabetes, heart disease, hypertension or hypercholesterolemia) using a survey previously used by us [13]. BMI (kg/m^2^) was determined from direct measurements of height and weight by study personnel. Eight participants who did not provide BMI remained in the analysis. During the intervention, participants recorded their daily physical activity, measured by pedometer (Yamax SW-200, Japan), in a calendar that was collected upon completion of the intervention. Data were collected from March through July, 2002 for the workplace groups and December 2002 through April 2003 in the community groups. Each participant provided 1–12 weeks of steps/day data (average 9.1 weeks or 64 days).

Weather for Charlottetown, Prince Edward Island (PEI) was recorded from publicly accessible databases maintained by Environment Canada [18]). Although study participants lived all across the province, it was assumed that the general weather characteristics would be similar because of PEI's small size (5,660 km^2^) [19] and the fact that the two major population centres in PEI are only 60 km apart.

### Statistical analysis

Demographic data, health and self-reported physical activity are presented as the proportion of participants for each categorical variable and as means ± SD for continuous variables. A mixed linear model was used to examine the relationship between activity (log steps/day) and summarized daily weather related variables: total rainfall (including rain, drizzle, freezing rain and hail in 24 h, mm), mean temperature (the average of the maximum and minimum temperature over 24 h, °C), total snowfall (including snow and ice pellets fallen in 24 h, cm), maximum wind speed (single peak speed recorded over a 24 h period, km/h), and accumulated snow on ground (depth, cm). Details of the weather data collection are found on the Environment Canada website [18]. A mixed model was used in order to handle the longitudinal error structure of a large number of individuals who were followed over time. It was assumed that their response to the intervention would vary among individuals and the mixed model allowed us to fit an "average" population response, and a set of random terms for each individual. The model controlled for BMI, gender, number of days on study ("days on study" is defined as the number of days each individual had been a participant in the intervention), weekday, month and the interaction between weekday and month. A first order auto-regressive error structure was used to account for possible correlations between adjacent serial observations within a subject. Multicolinearity between weather variables or between covariates and weather variables was not a factor in the analysis.

The final terms for the model were chosen using a backward model selection procedure with a Type I error rate of 5%. Non-significant terms were removed while maintaining a hierarchical model (i.e., if a quadratic term was required in the model, the lower order linear term was maintained regardless of the significance of the linear term) [20]. Representative outcomes obtained from the model were expressed as means ± 95% confidence intervals (CI).

## Results

### Participant characteristics

Demographic and health data of the participants are shown in Table 1. Females comprised 87.6% of the participants. The average BMI was in the overweight category (i.e. 25.0–29.9 kg/m^2^) [21] and the average steps/day was similar to that reported for other North American study groups of ostensibly healthy individuals [14,22]. Nine individuals declined to be measured for BMI and nine also declined to provide age data.

**Table 1:** Demographic and health characteristics of participants

**Parameter**	**Response**
Gender	
Male (count)	25
Female (count)	177
Age (years ± SD)	44.1 ± 9.9
Current smoker (%)	17.7%
% Diagnosed with...	
Heart disease	3.0%
Hypertension	17.2%
Hypercholesterolemia	12.3%
Type 2 diabetes	3.9%
Body mass index (kg/m^2^)	29.1 ± 5.7
Baseline steps/day (± SD)	7,635 ± 3,374

### Weather characteristics

Weather data for PEI (total rainfall, mean temperature, total snowfall, maximum wind speed and accumulated snow on ground) obtained from Environment Canada [18] are shown by monthly averages in Table 2. Winter months (December through March) temperatures were typically below 0°C and total snowfall ranged from 20 to 60 cm per month. Maximum wind speeds also tended to occur in the winter months.

**Table 2:** Weather characteristics by month in Charlottetown, PEI during 2002 and 2003

**Month**	**Mean Temperature (°C)**	**Total Rainfall (mm)**	**Total Snowfall (cm)**	**Maximum Windspeed (kph)**
*2002*				
March	-2.4	37.9	39.2	69
April	3.0	66.9	24.4	74
May	9.5	64.8	0.6	59
June	12.8	63.4	0	63
July	17.7	118.8*	0	72
December	-3.8	62.1	50.7	80
*2003*				
January	-9.9	6.2	59.0	74
February	-9.2	61.7	19.9	80
March	-4.4	93.9	50.8	76
April	1.9	47.4	24.8	76
May	8.0	64.4	0	50
June	15.4	85.0	0	54
July	19.9	57.8	0	56

*74.0 mm of rainfall was recorded on July 4, 2002. Because of this extreme value, data for that day have been removed from the analysis. Full details of the weather can be accessed at .

### Effects of day of week and month of year on physical activity

**Figure 1:**
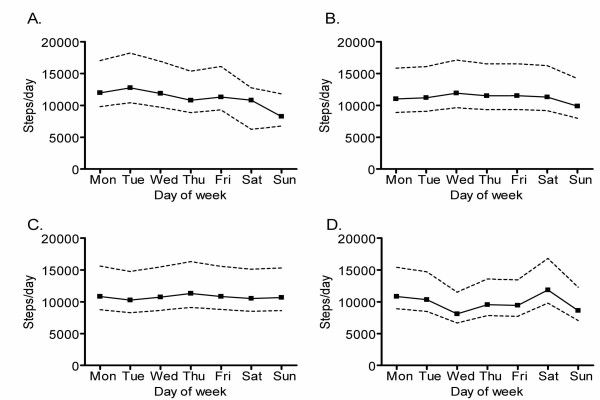
Weekly physical activity patterns as measured by pedometer in (A) February, (B) May, (C) July and (D) December. The pattern shown for February and May was similar to that observed in January, March, April and June (not shown). There were no data obtained for August-November. Steps/day on Sunday were significantly lower than the rest of the week in February and May. In July, there was no effect of weekday on steps/day. In December, steps/day were significantly higher on Saturday. Data are means ± 95% CI.

Anecdotal evidence from program participants suggested that day of week affected physical activity levels. In addition, we considered that month of year might also contribute to fluctuations in activity that were related to seasonal differences in physical activity and unrelated to daily weather fluctuations. The effects of weekday and month were found to interact (p < 0.0001), indicating that patterns of activity during the week were not independent of season. Data were available for December through July and four profiles representing the number of steps per weekday for four months are presented in Figure 1. The general trend for the first 6 months of the year were reflected in the data for February and May, where physical activity dropped by an average 25.6% (95% CI: 21.0%, 30.4%) on Sunday. This corresponded to a decrease of ~2,560 steps/day (2100 steps, 3040 steps) for an individual who took 10,000 steps/day on average. Exceptions to this trend appeared in July, where physical activity did not appear to drop significantly on Sunday compared with the rest of the week (the estimated drop was 1.1% (-15.8%, 11.7%). December was also an exception where physical activity was on average 18.1% (0.7%, 32.4%) higher on Saturday than the rest of the week (noting that physical activity did not differ from the rest of the week in either July, or a typical winter month (p > 0.05 in both cases). In addition, while physical activity on Sunday was 35.2% (9.8%, 66.7%) lower than physical activity on Saturday in December, there was a significant difference between physical activity on Sunday and the rest of the week (the estimated drop in physical activity was 14.6% (0.7%, 32.4%).

### Effects of weather on physical activity

The effects of weather indices and interactions with participant characteristics were reflected in the final model presented in Table 3. The most important result of the final model was that a variety of weather-related indices affected the activity of individuals, and in many cases the magnitude of the effect was related to personal characteristics such as BMI and gender. Results for each weather index and associated interactions are presented below.

**Table 3:** Final hierarchical model of factors affecting physical activity.

**Factor**	Type I P-value*	Type III P-value**
BMI	0.0273	0.0299
BMI^2^	0.0089	0.0136
Gender	0.5105	0.7973
Weekday	<0.0001	<0.0001
Month	<0.0001	0.0125
Weekday*Month	<0.0001	0.0002
Days On Study	0.0123	0.8453
Days On Study^2^	0.0442	0.0348
Days On Study^3^	0.0169	0.0056
Total Rain	<0.0001	<0.0001
Total Rain^2^	0.0019	0.0029
Mean Temperature	0.0002	0.0237
Total Snow	0.0541	0.0243
Total Snow^2^	0.4818	0.0394
Total Snow* Gender	0.0132	0.0129
BMI * Total Snow	0.5550	0.0227
BMI* Total Snow^2^	0.0375	0.0255
Maximum Wind Gust	0.0003	0.1455
Maximum Wind Gust^2^	0.0620	0.0211
BMI * MaximumWind Gust	0.9219	0.1089
BMI * MaximumWind Gust^2^	0.2046	0.0175
BMI ^2 ^* MaximumWind Gust	0.6069	0.0825
BMI ^2 ^* MaximumWind Gust^2^	0.0094	0.0112
Snow On Ground	0.0124	0.0124

* The Type I p-value is associated with sequential tests of hypothesis, thus order of the factor in the above table is important (see example below).

** The Type III p-value is associated with "last in" tests of hypothesis (see example below).

For example, consider the p-values for mean temperature. The Type I p-value measures the significance of the effect for mean temperature after adjusting for those effects in the table listed above mean temperature (BMI, Gender, Week, Month, Day on Study, Total Rain and the various interactions), but not those below mean temperature (Total Snow, Snow on Ground, and the various interactions). In contrast, the Type III p-value tests for the significance of the effect if it was the "last in" the model, i.e., adjusting for all factors in the table (BMI, Gender, Week, Month, Day on Study, Total Rain, Total Snow, Snow on Ground and the various interactions).

#### Total rain

**Figure 2:**
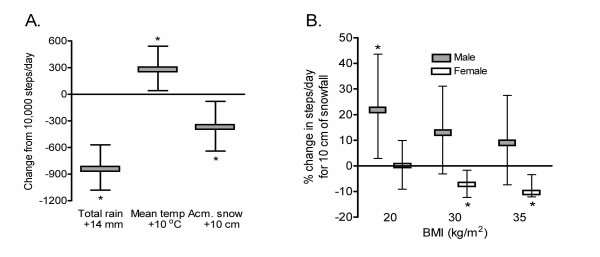
Effect of weather elements on physical activity determined from multivariable analysis. (A) Effect of total rain, an increase in temperature of 10°C and an increase of 10 cm in accumulated (accum.) snow on the ground on steps/day of an individual with a usual activity of 10,000 steps/day. No interaction with either BMI or gender was detected in multivariable analysis. (B) Percent change in steps/day of males and females, segregated into lean (BMI = 20 kg/m^2^), obese Class I (BMI = 30 kg/m^2^), or obese Class II (BMI = 35 kg/m^2^), in response to 10 cm snowfall in a 24 h period. Data are means ± 95% CI and *p < 0.05 or better.

The relationship between the log-transformed number of steps/day and a measure of the total amount of rain over 24 h was curvilinear in nature (p-value = 0.0029 after adjusting for all other factors in the model) and there was no evidence of significant interactions with either gender or BMI. In general, the number of steps/day decreased rapidly for small amounts of rain, and then flattened out. For example, the number of steps/day dropped by 5.2% (3.2%, 7.2%) over the first 5 mm of rain fall and reached a maximum decrease of ~8.3% (5.7%, 10.8%) at 14 mm of rain. Since there was no significant interaction between total rain and either gender or BMI, it appears that individuals were affected equally regardless of gender and BMI. Thus for an individual who took 10,000 steps/day, a rainy day (14 mm of total rain) reduced the number of steps/day by ~830 steps (570 steps, 1080 steps) (Figure 2A).

#### Mean temperature

A positive linear relationship occurred between steps/day and the mean temperature (p-value = 0.0237) after adjusting for all other factors. The net effect was ~2.9% increase (0.4%, 5.4%) in steps/day for every 10°C increase in mean temperature (Figure 2A). Thus for a person who took 10,000 steps per day, an increase in 10°C in any month resulted in an increase in physical activity of 290 steps (40 steps, 540 steps), independent of gender or BMI.

#### Total snowfall

The relationship between steps/day and the total snowfall was affected by both gender and BMI (p-value = 0.0129 and 0.0255, respectively, after adjusting for other terms in the model). The interaction between gender and total snowfall suggested a differential in steps/day between males and females that was related to the amount of snow that fell. Generally, as the amount of snow increased, males averaged more steps/day than females. For example, with a 10 cm snowfall, males took 16.7% (3.1%, 28.4%) more steps/day than females regardless of BMI in comparison with a 5 cm snowfall where men took 8.7% (1.6%, 15.4%) more steps/day.

The relationship between total snowfall and steps/day was also moderated by an individual's BMI (Figure 2B). For lean individuals (BMI = 20), the physical activity of males increased by 21.1% (2.7%, 43.4%) for the first 10 cm of snow. This increase was not seen for women with a BMI = 20 as physical activity increased by a non-significant 0.1% (-9.1%, 9.9%) for the first 10 cm of snow. For Category I obese male individuals (BMI = 30), there was no significant change in physical activity (12.7% increase (-3.1%, 31.1%)), while females with a BMI = 30 exhibited a significant 7.2% (1.7%, 12.3%) decrease in physical activity. For heavier individuals (BMI = 35), males exhibited a non-significant 8.5% (-7.4%, 27.2%) increase in physical activity, while females exhibited a significant 9.6% (2.5%, 16.2%) drop in physical activity. For an average lean male who took 10,000 steps/day, a 10 cm snow fall resulted in an increase in physical activity of 2,110 steps/day (270 steps, 4290 steps), while for an average lean female who took 10,000 steps/day, a 10 cm snow fall would result in no significant change in steps/day (-100 steps/day, -910 steps/day, 990 steps/day).

#### Maximum wind speed

Steps/day was affected by maximum wind speed and the BMI of the individual (p-value = 0.0112 after adjusting for all other factors), but did not appear to be related to gender. The relationship was quadratic in nature, and seemed to affect lean individuals the most. The decrease in the steps/day from a calm day (maximum wind gust of 40 kph) to a windy day (maximum wind gust of 60 kph) was the most for lean individuals (BMI = 20) at 5.2% (0.3%, 9.7%), intermediate for individuals with BMI = 35 at 3.4% (0.7%, 6.0%) and the least for individuals with a BMI of 30 at 2.8% (0.5%, 5.1%). The effect of wind speed was modest as a 20 kph change was required to bring about a change of between 2% and 5% in steps/day.

#### Accumulated snow on ground

The number of steps decreased by 3.6% (0.8%, 6.4%) for every 10 cm of accumulated snow on the ground, independent of gender and BMI (Figure 2A). Thus for an individual who took 10,000 steps/day on average, 10 cm of snow resulted in a decrease in physical activity by 360 steps (80 steps, 640 steps).

## Discussion

Many environmental factors contribute to an individual's willingness to engage in physical activity. Some factors, such as safety, aesthetic attributes and facilities [1] are unlikely to affect physical activity on a day-to-day basis. However, the weather, particularly in temperate climates, varies considerably by season and even day-to-day. Even within a given season, temperature, rain and snow, and maximum wind speed can vary markedly. In the current investigation, we found that individuals enrolled in an intervention designed to increase physical activity did have variations in day-to-day activity that were correlated with changes in the weather as well as day of the week and season.

Physical activity levels of PEI residents are among the lowest in Canada [23]. To address this, the PEI government supported implementation of a pedometer-based physical activity intervention that was delivered in two phases as pilot initiatives: first, in 5 workplaces and second, in communities across PEI. The outcomes of the workplace studies have been published [13,17] and those of the community intervention are ongoing (Chan and Tudor-Locke, unpublished data). Previously, pedometers were found to be useful for increasing physical activity by most study participants engaged in the FSP [24], and other pedometer-based programs [25]. The pedometer was likewise found to be useful for motivation and feedback in the workplace-based intervention conducted by us (Lauzon et al., unpublished data). For the purposes of physical activity research, pedometers are convenient, easy-to-use, accurate and inexpensive instruments for surveillance purposes [26].

The data collected in calendar format permitted us to study the effects of weather on the participants' physical activity levels by utilizing weather data available from Environment Canada. The analyses demonstrated that many aspects of weather potentially affected the steps/day accumulated by the study participants. Days on study was also a significant factor, which was predicted from our previous analysis showing that the intervention significantly increased physical activity [17]. Moreover, in some cases, the effect of weather indices (particularly total snowfall) was moderated by both the gender and the BMI of an individual.

Increasing daily temperature strongly predicted an increase in steps/day. Independent of the month, an increase in temperature of 10°C translated into a 2.9% increase in steps/day. For an average individual taking ~10,000 steps/day, this translates into an extra 290 steps/day. In PEI, there can be considerable day-to-day change in the mean temperature, particularly in the winter months. For example, in January 2003 the mean daily temperature ranged from -23.0°C to -0.3°C within one week. In March 2003 the range was -14.4°C to 5.3°C as recorded by Environment Canada over the whole month [18]. However, to put the 290 steps/day/10°C in context, the average number of steps taken in a 5 minute period ranges from 400–800 [27]; therefore these intervention participants probably met their intended physical activity goal for the day and the small decrease was not therefore likely to be clinically relevant. Although this study did not find a plateau in physical activity as the temperature became hotter, it seems plausible that extreme hot temperatures found in other geographic locations would be limiting to physical activity as was shown in the United Arab Emirates [28]. In contrast to a study of elderly Japanese, there was no plateau in physical activity at 17°C, which was considered to be thermoneutral [12]. Thermoregulatory capacity may decrease with aging, as shown by studies in humans [29] and rodents [30]; therefore, the physical activity of the younger population in our study (mean age 44 years) was probably less affected by temperature extremes.

Precipitation as rain negatively affected daily physical activity of our study population, as was shown by others in a Japanese group [12]. Interestingly, the decrement was approximately linear up to ~10 mm of rainfall but levelled off on wetter days. This suggests that regardless of rain patterns, a minimal amount of walking takes place, possibly related to access to indoor facilities for walking when the weather is poor. A rainfall of 14 mm would account for ~8.3% decrease in steps/day, which translates to 830 steps for an individual who averages 10,000 steps/day.

As many community-based programs were implemented in December 2002 through March 2003, precipitation in the form of snow was also found to significantly affect physical activity. The range in snowfall during that time frame ranged from 0 to 26 cm [18]. In fact, physical activity was affected by snowfall as well as the gender and BMI of the individual. In general, the more snow that fell, the more active men with lower BMI became. Accumulated snow on the ground also affected physical activity, independent of gender and BMI, by approximately 3.6% (or 360 steps/day) for every 10 cm of snow on the ground. For the majority of women in the study, who ranged in BMI from 20–35 kg/m^2^, snowfall had little effect on physical activity whereas men in the same BMI range increased physical activity. However, more than 10 cm of snowfall decreased physical activity of both men and women with a BMI >35 kg/m^2 ^and high average steps/day. Once again, the causes of the differences in steps/day associated with BMI and gender can only be speculated upon. However, it might be that leaner men assume snow-removal duties to a higher degree than women or obese men. Increasing snowfall might increase the fear of falling, perhaps more so in obese than lean individuals. Regular physical activity can decrease the risk of falling [30] yet a fear of falling is pervasive and negatively affects participation in physical activity, at least in the older, healthy women in whom it has been studied [32]. Whether there is an interaction between obesity and fear of falling is not known. Interestingly, however, mathematical modelling suggests that individuals with abdominal obesity have greater postural instability and may be at higher risk of falling [33].

Interestingly, maximum wind speed was also related to a decrease in physical activity, although this decrease was modest as a 20 kph increase was required to elicit a negative change in activity of 2 to 5%. Wind speed was not a significant factor in Japanese study but the wind speeds reported in that study (1–4 m/s or <20 kph) [12] were much lower than those to which the PEI participants were subjected on a regular basis. Therefore, precipitation (i.e., rain and snow) and temperature appear to be more important factors that influence physical activity than wind speed. However, wind speed might emerge as a more consistent factor if mean wind speed over 24 h was available, rather than peak wind speed within a 24 h timeframe. For example, if the peak wind speed occurred during the night yet the day was relatively calm, little effect on physical activity would be predicted, assuming that most participants in the study walked during the day.

The day of the week has also been shown to contribute to day-to-day variability in physical activity measured by pedometer [11]. Physical activity measured on Sunday is significantly lower than other days of the week [11]. In other analyses, self-report of physical activity on Saturday and Sunday correlated poorly with activity levels on other weekdays [34,35]. Our data support the accumulating evidence that Sunday remains a "day of rest" for many people. However, we also noted some seasonal variation in activity patterns. The peak activity on Saturday during December was interesting because at the time of data collection in 2002, Sunday shopping was not permitted in PEI, so Saturday likely represented peak shopping behaviors. It would be of interest to see whether this pattern has changed since the introduction of Sunday shopping in December in 2003. Moreover, the relatively consistent activity from day-to-day recorded in July might reflect either greater consistency in the weather or the possibility that many people were taking vacation during that time, which would eliminate any work-related variability in physical activity.

The strength of this study was that objective data of both physical activity (pedometer-determined steps/day) and weather were utilized to determine the significant factors. Previous studies have relied upon self-report of individuals as to the importance of weather with regards to physical activity behaviours [1]. Our data showing that fluctuations in daily temperature became less influential on physical activity as the days on study increased is consistent with self-report data showing that men who were "high exercise" walkers or women who were "high neighborhood" walkers were less likely to perceive the weather as a deterrent to walking [2]. This implies that people with a strong commitment to physical activity, including those enrolled in interventions like the participants in the current study, are willing to accept some unpleasant weather. Although temperature, rain and wind did have a significant effect, the actual changes in activity were relatively small. In two smaller studies of individuals not in any intervention, season of the year did affect physical activity [11,12]. Interestingly, the individuals in those studies lived in South Carolina and Tennessee in the United States or in Japan, where seasonal variations in climate extremes are less marked than in PEI. It would be of interest to conduct a larger study to compare effects of weather on individuals in an intervention versus those who are not to substantiate our hypothesis that sufficiently motivated individuals are less affected by weather conditions than those who view physical activity as only one option of many for their leisure time. In addition, to more fully understand differences between genders, larger samples of men are required.

One limitation of this study is that the participants were pooled from two separate arms of the FSP intervention. In the community based-program, many participants enrolled in the December to March period when snow was an important variable whereas the workplace participants commenced their programs in March through May. The seasonal difference may have been a confounder, but both month and the interaction between month and day-of-week were controlled for in the analysis. Another limitation is that the participants volunteered for the intervention and were more-or-less motivated to achieve self-selected physical activity goals; as noted above, this may decrease weather-related effects on activity. Moreover, PEI has a largely rural population so it is unclear whether the findings apply to more urban populations.

## Conclusion

Understanding the environmental factors that impact on physical activity is important for program coordinators and policy makers. To date, there have been only a few studies that address these factors, including weather, and only two small investigations utilized an objective measure of physical activity in a longitudinal design [11,12]. In our study of more than 200 individuals, in which daily activity and weather data were both objectively determined, we have shown for the first time the potential for variables such as temperature, rain and snow, and wind speed to alter daily physical activity. Further work should be aimed at examining physical activity among the general population correlated with weather variables.

## Abbreviations

BMI, body mass index; PEI, Prince Edward Island; CI, confidence intervals.

## Authors' contributions

CBC and CT conceived of the study and participated in its design. In addition, CBC oversaw completion of the study and drafted the manuscript. DAJR performed the statistical analysis. All authors contributed to writing and approved the final manuscript.
